# Kinematic analysis of diastolic function using the freely available software Echo E-waves – feasibility and reproducibility

**DOI:** 10.1186/s12880-016-0162-8

**Published:** 2016-10-27

**Authors:** Martin G. Sundqvist, Katrin Salman, Per Tornvall, Martin Ugander

**Affiliations:** 1 Department of Clinical Science and Education, Södersjukhuset, Karolinska Institutet, and Cardiology Clinic, Södersjukhuset, SE-188 83 Stockholm, Sweden; 2 Department of Clinical Physiology, Karolinska Institutet, and Karolinska University Hospital, SE-171 76 Stockholm, Sweden

**Keywords:** Diastolic function, Echocardiography, Kinematic analysis, Software

## Abstract

**Background:**

Early diastolic left ventricular (LV) filling can be accurately described using the same methods used in classical mechanics to describe the motion of a loaded spring as it recoils, a validated method also referred to as the Parameterized Diastolic Filling (PDF) formalism. With this method, each E-wave recorded by pulsed wave (PW) Doppler can be mathematically described in terms of three constants: LV stiffness (*k*), viscoelasticity (*c*), and load (*x*_0_). Also, additional parameters of physiological and diagnostic interest can be derived. An efficient software application for PDF analysis has not been available. We aim to describe the structure, feasibility, time efficiency and intra-and interobserver variability for use of such a solution, implemented in Echo E-waves, a freely available software application (www.echoewaves.org).

**Results:**

An application was developed, with the ability to open DICOM files from different vendors, as well as rapid semi-automatic analysis and export of results. E-waves from 20 patients were analyzed by two investigators. Analysis time for a median of 34 (interquartile range (IQR) 29–42) E-waves per patient (representing 63 %, IQR 56–79 % of the recorded E-waves per patient) was 4.3 min (IQR 4.0–4.6 min). Intra-and intraobserver variability was good or excellent for 12 out of 14 parameters (coefficient of variation 2.5–18.7 %, intraclass correlation coefficient 0.80–0.99).

**Conclusion:**

Kinematic analysis of diastolic function using the PDF method for Doppler echocardiography implemented in freely available semiautomatic software is highly feasible, time efficient, and has good to excellent intra-and interobserver variability.

## Background

Diastole, the time during which the ventricles of the heart fill with blood, is conventionally assessed by measuring various blood flow velocities, time intervals, ratios of velocities, and dimensions of chambers and walls of the heart [1]. The investigator then tries to fit these measurements with a theoretical and empirical body of knowledge constituting part of our understanding of left ventricular (LV) mechanics and hemodynamics. As such, the conventional method of assessing diastolic function lacks a unifying theory. Improving our ability to investigate diastolic function is of importance not only as it is vital to our understanding of cardiac function in health and disease, but also in order to better approach diagnostics and treatment options in the increasingly recognized condition of heart failure with preserved ejection fraction. We aim to describe an alternative strategy for assessing diastole, and a software implementation for this method that is ready for use in clinical research.

During the cardiac cycle, the LV contracts during systole, whereby the volume of blood in the LV decreases. During early diastole, the LV recoils, leading to an increase in the volume of blood in the LV, with blood entering the LV through the mitral valve. In the absence of aortic regurgitation or shunts, the velocity of blood flow through the mitral valve during early diastole will be the same as the velocity of volume expansion of the LV, and hence the velocity of LV recoil. The velocity of transmitral blood flow can readily be measured by pulsed wave (PW) Doppler echocardiography. It has been shown that the velocities of early diastolic transmitral blood flow, and thus LV recoil, can be accurately described as a case of damped harmonic motion [2]. Damped harmonic motion is a part of the analytical framework describing motion in classical mechanics, and can been used, among other things, to describe the recoil of a spring. From this framework one can derive a mathematical expression that describes velocity as a function of time in terms of three constants, namely stiffness (*k*), viscoelasticity, or energy loss, (*c*), and load (*x*_0_). By curve fitting the velocity profile of the PW Doppler signal of the E-wave to the mathematical expression, the constants can be obtained. The theoretical framework describing LV filling in this manner has been termed the parameterized diastolic filling (PDF) formalism [2]. Using gold standard high fidelity invasive hemodynamic measurements, the stiffness constant *k* has been shown to have a close linear relationship with LV diastolic stiffness [3]. In the same manner, it has been shown that the influence of the damping constant *c* can be used to formulate an accurate estimate of the invasively determined time constant of isovolumic pressure decay, *tau* [4]. Furthermore, a load independent index of diastolic filling, called *M*, can be calculated by examining the relationship between the peak driving force and the peak resistive force of filling at different levels of load [5]. Furthermore, clinical studies have illustrated the utility of the PDF method in characterizing clinical disease including diabetes [6, 7], hypertension [8], as well as prognosis in congestive heart failure [9]. A brief overview of the PDF parameters is given in Table 1, and further details are given in the Appendix. In summary, this unified approach to studying early diastolic filling narrows the gap between clinical echocardiography and classical mechanics, and also promises new insights into different pathological states as well as basic cardiac physiology. However, a user-friendly solution enabling PDF analysis of echocardiographic DICOM images and output of results in a fashion optimized for clinical research use has not been available [10]. Therefore, we aim to describe the structure, feasibility, time efficiency and intra-and interobserver variability for use of such a solution, implemented in Echo E-waves, a freely available software application.

**Table 1:** Overview of PDF parameters

Parameter	Name	Unit (SI unit)	Physiological description
*x*_0_	Load	cm	Related to the load that is compressing the elastic myocardium at end systole, a prerequisite for a restoring force to arise. Closely related to the velocity time integral (VTI) of the E-wave.
*k*	Stiffness	g/s^2^ (N/m)	LV rigidity, or the extent to which the LV resists deformation in response to an applied force. Linearly related to chamber stiffness [3] (dP/dV), and thus influences the restoring force that drives early diastolic filling. Increased in hypertension [8].
*c*	Viscoelasticity	g/s (N∙s/m)	Energy loss or damping of LV recoil, caused by impaired relaxation and increased viscoelasticity of the myocardium. Increased in diabetes [6] and hypertension [8].
Vmax	E-wave peak velocity	m/s	Peak velocity of blood flow across the mitral valve during early LV filling.
*kx*_0_	Peak driving force	mN	The peak force driving LV filling, analogous to the peak atrioventricular pressure gradient [14]. The product of *k* and *x*_*0*_,
*c*Vmax	Peak resistive force	mN	The force resisting filling at peak transmitral flow. The product of *c* and Vmax.
1/2*kx*_o_^2^	Filling energy	mJ	Stored potential elastic energy from systole that generates rapid early LV filling. Increased in hypertension [8].
*c* ^2^ − 4*k* or β	Damping index	g^2^/s^2^ (kg∙N/m)	Reflects the balance between the factors driving and resisting left ventricular filling. Values < −900 g^2^/s^2^ are a strong predictor of 1-year mortality in elderly with heart failure [9].
KFEI	Kinematic filling efficiency index	%	An index that characterizes the efficiency of LV filling. Calculated as the ratio of the velocity time integral of the actual E-wave to the velocity time integral of a PDF model-predicted ideal E-wave contour with no resistance to filling (*c* = 0) but the same stiffness (*k*) and load (*x*_0_) as for the original E-wave. Reduced in diabetes [7].
tau	Time constant of isovolumic pressure decay	ms	Tau is used to characterize LV filling based on time-resolved high fidelity invasive measurements of LV pressure. Increased in impaired relaxation. Can be approximated by combination of PDF parameters [4].
M	Load independent index of diastolic filling	unitless	A load independent index of diastolic filling, which is decreased in patients with diastolic dysfunction and increased LV end-diastolic pressure [5]. Describes the ratio of change in peak driving force to change in peak resistive force (Δ*kx*_0_/Δ*c*Vmax), calculated after acquiring E-waves under varying loading conditions.
B	Intercept	mN	An index of diastolic filling that is increased in patients with diastolic dysfunction and increased LV end-diastolic pressure [5]. Mathematically, the y-axis intercept of the relationship between the peak driving and resistive forces from E-waves acquired under varying loading conditions.

*PDF* parameterized diastolic filling, *LV* left ventricle

## Implementation

The program, Echo E-waves, is freely available for download at www.echoewaves.org [11]. It is implemented in the MATLAB® release 2015a (Mathworks, Natick, Massachusetts, USA) environment for Windows (64-or 32-bit architecture). In the following section we describe the structure and functionality of the program. For analysis of inter-and intraobserver variability, sets of E-waves with visually moderate to good quality were selected from 20 patients undergoing echocardiography as part of ongoing clinical studies on adults with acute myocardial infarction with or without obstructive coronary artery disease, and excluding patients with significant valvular disease. The studies were approved by the Regional Ethical Review Board in Stockholm, Sweden, and all subjects provided written informed consent. All echocardiographic acquisitions were performed on Vivid E9 scanners (GE, Horten, Norway). The Echo E-waves program was run on an Intel® Core i5™ 2500 K processor with a NVIDIA® GeForce® GTX 670 graphics card and the 64-bit version of Windows 7.

### Echocardiographic image acquisition

The program can analyze PW Doppler echocardiographic images of transmitral flow acquired in the same manner as is recommended by clinical guidelines [12]. However, some adjustments are suggested to ensure improved accuracy in analysis. A horizontal sweep speed of 100 mm/s is recommended, as lower sweep speeds lead to a substantial loss of temporal resolution. A sweep speed of 150 mm/s or 200 mm/s is also acceptable. However, in our experience these sweep speeds make the visual recognition of the shape of the E-wave somewhat more difficult, since they are less commonly used clinically. The program currently reads DICOM images from GE, Philips, Siemens and Acuson systems. When using a GE or Philips system, the user can export a cine recording of Doppler data stretching over several screen widths as a single DICOM multi frame file. A method for this is described in on the program website (www.echoewaves.org). From all the systems above, single frame DICOM image files can also be loaded. For the purposes of testing the intra-and interobserver variability, 20 echocardiographic exams collected during ongoing clinical trials were selected. The exams were performed by four dedicated sonographers, all with at least 8 years of experience with clinical echocardiography. A 3.5 mm sample volume was placed at the tips of the mitral leaflets, and PW Doppler recordings were performed both during free breathing and after cessation of the Valsalva maneuver.

### Loading and visualization of Doppler data from DICOM images

**Fig. 1:**
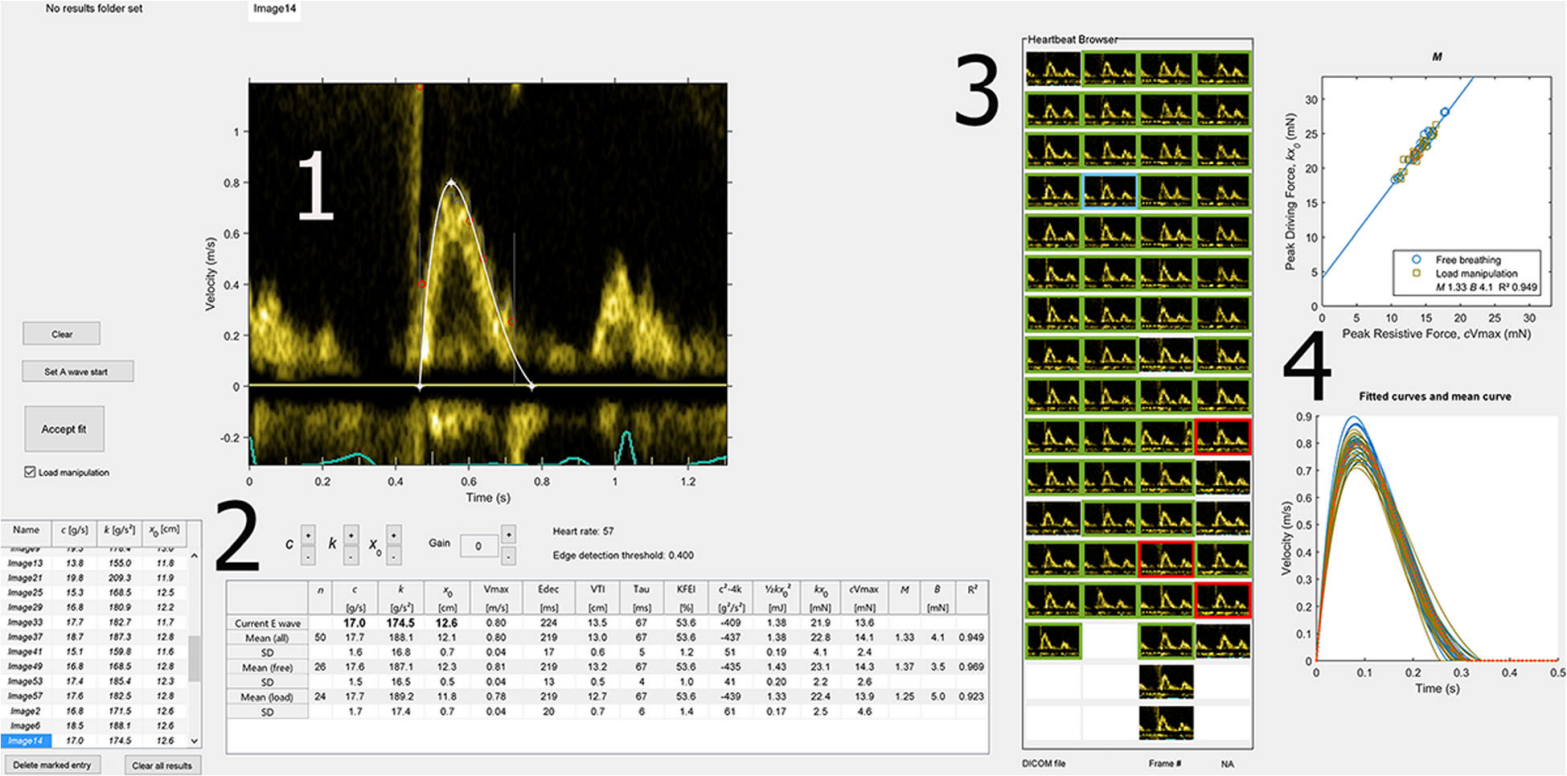
Overview of the graphical user interface. **1**: Display of Doppler data, velocity profile detection and fitted *curve* for a given heart beat. **2**: Numerical display of results, per E-wave (list on *left*) and summarized (table on *bottom right*). **3**: Overview of each E-waves in the current acquisition comprised of multiple E-waves. **4**: Graphs of (*top*) the basis for calculation of the load independent index of filling, *M*, and (*bottom*) all accumulated PDF curves for each E-wave

Using DICOM metadata, the Doppler region of the image is automatically identified and displayed with correct time and velocity scaling. For ease of overview, the contents of the folder are displayed as thumbnails in the Heartbeat Browser, also enabling navigation of the data set using mouse or keyboard input. Extended (cine) recordings of Doppler registrations from GE and Philips scanners are automatically cropped to one image per cardiac cycle. In order to facilitate the detection of the part of the Doppler signal constituting the E-wave, the user can change color maps, gain settings and apply contrast stretching. Currently, the program does not load the contents of folders with a mix of single frame and cine DICOM files. The graphical user interface is described in Fig. 1.

### Detection of the velocity profile

In the data structure of a DICOM image, Doppler data is represented as a two dimensional matrix of numbers, with each matrix index corresponding to a velocity at a given point in time. In these images, the horizontal axis of the image is time in seconds, and the vertical axis is velocity in meters per second. The value at a given matrix index indicates the brightness of the pixel at the corresponding position in the image, and the brightness corresponds to the amount of blood travelling at that given velocity at that point in time. For the purpose of detecting the velocity profile, the program searches each column (i.e. each time point) of the image from the top down for the first pixel having a brightness higher than or equal to a user defined threshold level, matching each time point with a velocity. However, as the Doppler recording is susceptible to noise, and due to the presence of non E-wave signal, the algorithm has been optimized by taking into account pre-existing knowledge of how the velocity profile of the E-wave can be differentiated from Doppler signal not originating from the E-wave. For instance, velocity at the moment when flow is about to begin must be zero. Furthermore, we can assume that there must be an upper limit to the velocity difference between two time points a few milliseconds apart. The algorithm accordingly discards a detected velocity that differs too much from the previous velocity. Discarded velocities are displayed for reference and transparency as to the behavior of the algorithm.

The user can adjust the input to the curve fitting algorithm with regards to the time points along the horizontal axis of the Doppler image at which the detection algorithm starts and ends, as well as the threshold of Doppler signal intensity at which detection occurs. Notably, it is of great importance that the starting point is set accurately at the onset of flow, as a small deviation can lead to substantial differences in the constants resulting from curve fitting. In many cases, the exact position of the onset of flow can be difficult to ascertain visually at first glance. However, the velocity profile during the acceleration time is often quite distinct, and if close attention is paid to how different starting points affect the visual correspondence of the PDF curve to the underlying Doppler data of the acceleration time and the upper, often well defined part of the E-wave, a convincing position for the starting point can be identified in effectively all cases. Positioning of the end point must take into account the various non-E-wave Doppler signals that frequently occur during the deceleration phase. A good curve fit will often be achieved if the end point is set at approximately 75 % of the total E-wave duration and typically at less than 50 % of the maximum E-wave velocity. In the case of partial overlap of the E-and the A-wave, the user must examine the E-wave to see if it is of sufficient duration to determine where it would have ended had there been no overlap between the E-and the A-wave. Typically, this is not possible when the crossover point between the E-and the A-wave occurs at a velocity is above 50 % of the maximum velocity of the E-wave. The program contains no particular feature to guide the user in this process, but using the alternative method to curve fitting described below can often make the task easier. We recommend excluding E-waves from analysis when there is an overlap with the A-wave at greater than 50 % of the maximum velocity of the E-wave. To further increase the agreement between the final fitted curve and the E-wave portion of the Doppler signal, the program also has two options that will make the deceleration time of the fitted curve shorter. The rationale for this is that the various artifacts and non-E-wave blood flow signals during the deceleration phase usually have higher velocities than the true E-wave, and thus will prolong the deceleration time of the fitted curve if they are included. The first approach to alleviate this is to simply replace the final two detected velocities with the value zero. The second approach is to first fit a curve to the detected velocities, as described below, and then calculate the tangent at the time point where the velocity has decreased to 70 % of the maximum velocity. Extrapolating this tangent to the velocity baseline will form a close approximation of the line drawn in clinical echocardiography to mark the deceleration time. This straight line of velocities is then substituted for the velocities of the original fitted curve during the corresponding time frame, and this new set of velocities is then fitted again, producing a final curve with a shorter deceleration time. On a modern computer, these calculations are performed virtually instantaneously. By default, both approaches for making the deceleration time agree more with clinical practice are enabled. It should be noted that these two alterations of the curve fitting process, although having justifications in physiology (if the E-wave ends, its velocity must be zero at the end) and the nature of the Doppler acquisition (noise and non-E-wave signal during the deceleration time and diastasis), they are based on empirically determined parameters. In our experience, using them will lead to a faster and more intuitive curve fitting workflow, in that the sensitivity to the problems mentioned above will be reduced. These fitting settings should be viewed as improvements to speed of effectively obtaining a visually acceptable fit, and not as changes to the PDF model per se. If the user so chooses, they can be disabled individually.

### Curve fitting and adjustment of the fitting algorithm input

The paired time-velocity data obtained during detection is used as input to a native curve fitting function of MATLAB, which employs the Levenberg-Marquardt algorithm, a standard algorithm for solving non-linear least square problems. After curve fitting, the constants *c*, *k*, and *x*_0_ are obtained with values that, when used as input to an expression of velocity as a function of time, would yield the closest match to the originally detected velocity profile. This calculated curve is displayed as an overlay to the original Doppler image, along with the result of the velocity profile detection algorithm for visual confirmation. After display of the fitted curve, the user can adjust the start and end points of the velocity profile detection time interval, as well as the threshold for detection, resulting in an automatic update of the fitted curve. These adjustments can be made by clicking and dragging with the mouse, or by using keyboard shortcuts. No measures of goodness of fit for the E-wave are displayed. Mathematically, the curve fitting process will produce an excellent fit in nearly all cases, and differences in e.g. sum of squared errors cannot be used to decide if an adjustment to a fit will result in a physiologically more accurate fit. Rather, we have found that visual assessment is the most reliable and useful method for assessing the appropriateness of a given fit.

### Automatic detection and curve fitting

In order to facilitate curve fitting of data sets comprised of large numbers of E-waves, a semi-automatic algorithm for curve fitting several E-waves has been implemented. The user must first set the starting point and adjust the settings so that the first E-wave is fit in an acceptable manner. The program stores the part of the image corresponding to the start of this user-defined E-wave as a template. The template is then used to find the starting point of the E-wave on the subsequent images using normalized cross correlation. The velocity profile detection algorithm described above is then run from this starting time point to either 1/3 of a cardiac cycle, or to the time point where velocity has fallen below 35 % of the peak velocity of the E-wave. The detected velocities are used for curve fitting as described above. The resulting parameters are compared to the mean of the previously obtained parameters, and if any parameter of the current E-wave deviates more than 40 % from the mean of the previous, the program discards the fit and moves on to the next E-wave. Usage of this automated method can speed up analysis, however, it requires close scrutiny of the obtained results.

### Quality control

In order to review produced results, and also facilitate the identification of anomalies, the results are displayed simultaneously in multiple fashions. The numerical values of the constants *c*, *k*, and *x*_0_ are displayed in a list, and the mean of the constants and the derived parameters are also displayed. Furthermore, the calculated curve for each E-wave is displayed in a graph, where each curve is selectable. Each E-wave is also displayed as a selectable marker in a plot of peak driving force vs. peak resistive force. Selecting an E-wave in any of these displays retrieves and displays the corresponding E-wave with its fitted curve, which can then be adjusted or deleted if the user so chooses.

### Alternative method to curve fitting

It has been shown that the curves yielded by applying the mathematics of damped harmonic motion to Doppler E-wave velocity profiles can be fully described in terms of unique combinations of their acceleration and deceleration times and peak velocities [13]. In other words, each unique combination of the constants *c*, *k*, and *x*_0_ also has a unique combination of acceleration and deceleration time and peak velocity. As a consequence of this, the constants for an E-wave can be calculated if these two time intervals and the velocity are known. As an alternative to fitting the full data set of velocities, it is thus possible to use just these three measurements. In the program, this is implemented both as a primary way of producing a curve, and as a method for adjusting a previously obtained curve. The user can produce a curve by marking the start, peak velocity and end points. For any curve, regardless of if it was produced by curve fitting or this alternative method, the start, peak and end points are manually adjustable by dragging them with the mouse, enabling an easy user interface for adjusting the shape of the curve, which is updated in real time.

### Export of data

All constants and derived parameters can be saved and exported to a spreadsheet. The Appendix details the mathematical calculations employed in the software for subsequent calculation of derived parameters. If Excel® is installed on the computer on which Echo E-waves is running, the results can be saved in.xlsx format, and otherwise in a comma separated values.txt format. Saving of images and graphs is also possible. It is also possible to copy the results to the computer’s memory clipboard and paste them into a program of choice. The program also stores the part of the DICOM images containing the Doppler data as well as any results of curve fitting and user interaction, so that images and/or curve fits for a given set of DICOM files can be retrieved, reviewed and/or edited at a later time. Saving in this manner also enables full optional anonymization, leaving the saved file without any trace of patient or source file identification. These data files are created in the native .mat format of MATLAB. When saving the results of an analyzed case, it also possible to designate a results folder, which will save all results to a common space, and also append each case to a file (.xlsx or.txt) with the means and standard deviations of all constants for each case, effectively creating a study database.

### Statistical analysis

All statistical analyses were performed in MATLAB release 2015a (Mathworks, Natick, Massachusetts, USA). The basic PDF constants and the derived parameters were analyzed as mean values for each patient. The coefficient of variation (CV) for the difference between measurements, intraclass correlation coefficient, and percentage difference with standard deviation were calculated and presented as needed. The CV was calculated as the standard deviation of the mean differences between analyses divided by the mean and expressed as a percent. Durations of analysis and numbers of analyzed E-waves are reported as median and interquartile range (IQR).

## Results

**Fig. 2:**
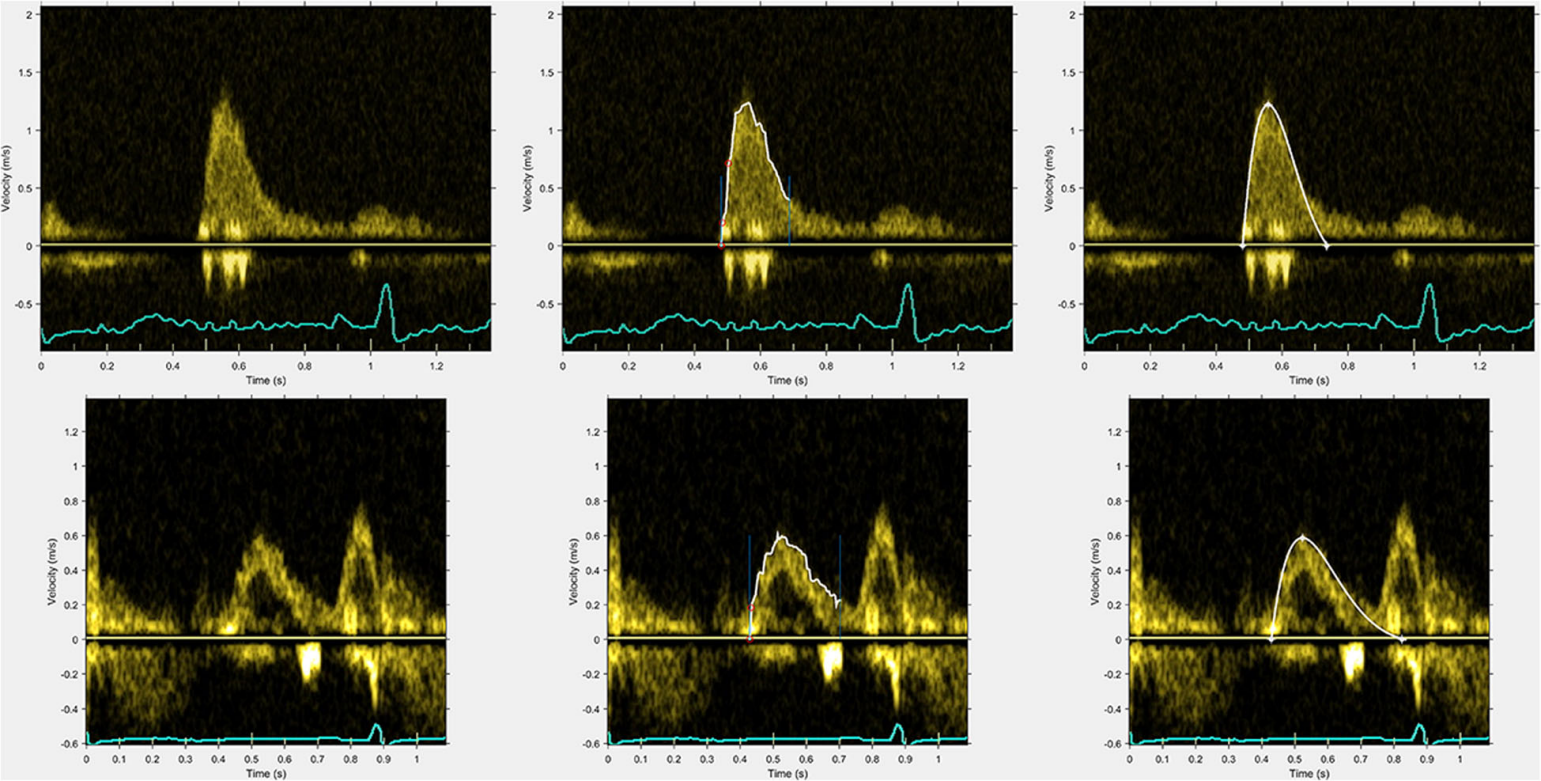
Representative examples of Doppler velocity profile edge detection and the resulting fitted *curves* for two different patients (*top* and *bottom*, respectively). *Left* panels: original Doppler images. *Mid* panels: detected edges of the velocity profiles in *white*. *Right* panels: fitted *curves* (*white*). PDF parameters for the *top row*: *c* 17.3 g/s, *k* 135 g/s^2^ *x*_0_ 11.4 cm; *bottom row*: *c* 17.7 g/s, *k* 226 g/s^2^, *x*_0_ 16.0 cm

Representative results of the performance of the velocity profile detection algorithm and the corresponding fitted curves are shown in Fig. 2.

For each patient, 34 (29–42) E-waves were analyzed. Thus, 63 (56–79) % of the recorded E-waves per patient were deemed to be of sufficient quality to perform curve fitting with visually acceptable results. The time for analyzing one patient case was 4.3 (4.0–4.6) minutes. These timings were calculated by letting the automatic fitting algorithm analyze all E-waves, after which the investigator reviewed the whole set, making adjustments as necessary. The median, range and interquartile range of the analyzed parameters are shown in Table 2. For the peak driving force, the per patient mean range was 8 mN.

**Table 2:** Results of PDF analysis in 20 patients

	Median	Range	Interquartile range
*c* (g/s^2^)	17.4	10.6–32.8	15.5–20.4
*k* (g/s)	177.4	106.5–294.8	153.9–206.9
*x*_0_ (cm)	11.7	7.7–17.9	10.3–12.8
Vmax (m/s)	0.7	0.5–1.3	0.6–0.8
Edec (ms)	236	139–473	205–273
VTI (cm)	12.4	8.1–19.1	11.0–13.5
Tau (ms)	70.1	47.6–151.6	62.0–83.3
KFEI (%)	53.0	46.1–64.1	51.4–54.8
*c* ^2^ − 4*k* (g^2^/s^2^)	−382	−224– − 826	−295– − 474
Filling energy (mJ)	1.1	0.6–3.3	0.9–1.4
Peak driving force (mN)	19.6	12.4–40.6	17.6–22.7
Peak resistive force (mN)	12.5	7.6–27.3	10.6–14.7
M (dimensionless)	1.17	0.96–1.49	1.06–1.26
B (mN)	5.3	1.6–9.7	4.1–6.7

*Vmax* E-wave peak velocity, *Edec* E-wave deceleration time, *VTI* velocity time integral, *KFEI* kinematic filling efficiency index

### Inter-and intraobserver repeatability

The results for observer variability are presented in Table 3. Overall, inter-and intraobserver reliability was good to excellent. The investigators were free to discard E-waves for which a visually convincing fit was not possible to obtain. Limiting analysis to only those E-waves for which both investigators had accepted fits yielded similar results. The notable exception to this was the parameters *M* and *B*. For *M*, interobserver variability was high when all fitted E-waves were considered. Discarding those E-waves for which one of the investigators had not provided a result yielded a substantial improvement. By comparison, the interobserver variability for *B* was substantial using both approaches.

**Table 3:** Inter-and intraobserver variability

3A	Intraobserver				Interobserver			
	CV (%)	Difference (%)	SD	ICC	CV (%)	Difference (%)	SD	ICC
*c*	13.2	12.6	9.8	0.80	11.6	9.8	6.1	0.92
*k*	9.3	9.1	7.3	0.93	14.0	9.0	8.3	0.88
*x*_0_	6.6	4.7	4.8	0.95	5.9	4.7	4.6	0.96
Vmax	5.1	3.3	4.0	0.98	4.0	3.0	2.6	0.99
Edec	11.1	7.6	7.5	0.92	11.8	9.1	6.2	0.93
VTI	6.0	4.1	4.2	0.96	5.3	4.3	3.4	0.97
Tau	11.7	8.9	8.4	0.88	13.3	10.2	6.7	0.91
KFEI	2.7	2.6	2.0	0.89	2.5	2.2	1.9	0.91
*c* ^*2*^ − 4*k*	18.7	18.9	30.1	0.92	14.3	16.2	14.7	0.95
Energy	14.6	13.7	9.6	0.94	10.6	9.8	6.4	0.98
Peak driving force	10.7	9.8	8.1	0.92	10.6	8.1	6.0	0.95
Peak resistive force	14.6	13.5	10.6	0.85	12.9	10.6	7.2	0.93
M	15.3	12.1	8.3	0.51	23.7	17.7	20.8	0.16
B	44.7	39.5	32.6	0.37	51.1	37.0	38.7	0.32
3B	Intraobserver				Interobserver			
	CV (%)	Difference (%)	SD	ICC	CV (%)	Difference (%)	SD	ICC
*c*	13.6	12.5	10.6	0.80	11.6	10.4	5.3	0.93
*k*	9.0	8.6	6.8	0.94	13.7	9.2	7.7	0.88
*x*_0_	5.5	3.9	4.8	0.96	5.5	4.3	4.5	0.96
Vmax	2.4	2.3	1.9	0.99	3.8	3.0	2.5	0.99
Edec	9.7	6.9	7.1	0.94	11.5	7.9	6.6	0.94
VTI	4.0	2.9	3.1	0.98	5.1	4.2	3.2	0.97
Tau	10.7	8.6	8.0	0.90	13.1	8.8	7.1	0.92
KFEI	2.9	2.6	2.3	0.88	2.6	2.4	1.8	0.91
*c* ^2^ − 4 *k*	17.9	15.8	25.0	0.93	12.7	13.1	11.9	0.96
Energy	16.0	14.2	11.0	0.93	10.9	9.7	6.5	0.98
Peak driving force	11.0	10.5	8.5	0.91	10.8	8.2	5.9	0.94
Peak resistive force	15.7	14.5	11.8	0.83	13.3	10.9	6.9	0.93
M	15.4	12.1	8.3	0.61	13.5	10.2	11.2	0.71
B	44.7	39.5	32.6	0.51	27.9	19.6	19.9	0.83

3A are the results obtained when observers were free to choose which E-waves to analyse from each set. 3B are the results obtained when comparing only those E-waves which both observers had analyzed. For *c* ^2^-4 *k*, the coefficient of variation (CV) and percentage difference are given as positives for ease of comparison, although they are negative values mathematically. *Vmax* E-wave peak velocity, *Edec* E-wave deceleration time, *VTI* velocity time integral, *KFEI* kinematic filling efficiency index

## Discussion

We have described how the freely available software application Echo E-waves can be used to analyze transmitral Doppler recordings in the PDF framework. The application can be used to obtain results in a timely fashion, and makes it feasible to investigate further usage of this approach to measuring and assessing diastolic function. Furthermore, intra -and interobserver reliability was good, with the exception of the load independent index of filling, *M*, and the intercept *B*. There may be several reasons for these findings. As it is defined, *M* is the slope of a regression line describing the relationship between driving and resistive forces under different loading conditions. As for all linear regression slope values, the accuracy of *M* can be disproportionately influenced by outliers, and the decision to include a particular E-wave can have a sizable impact on *M*. In order to alleviate the disproportionate impact of potential outliers, one would prefer a data set with a wide variation in load, with a roughly equal spread of E-waves with regards to differences in loading conditions. In our population, using the load variation after the Valsalva maneuver, the per patient mean range of peak driving force was only 8 mN. By comparison, in the study by Shmuylovich *et al*, the range was 30–40 mN, with load variation induced by varying the body position of the subjects using a tilt board and body positions ranging from head up 90° to head down 90° [5]. It seems likely that this larger load variation could lead to a more accurate assessment of *M*, however, the present study is the first to analyze the inter-and intraobserver reliability regardless of loading conditions. It should also be noted that the optimal cut off values and ranges for using *M* for clinical or scientific work have yet to be determined. Other methods for inducing variations in load are worthy of exploration. Furthermore, the optimal number of E-waves necessary for reliable analysis is also worthy of further investigation. It should also be noted that we did not examine the test-retest variability. Future studies incorporating multiple examinations per subject are motivated in order to address the effects of user dependency in echocardiographic image acquisition on quantification of PDF measures.

To our knowledge, there is only one other publicly available software application for PDF analysis [10]. The advantages of Echo E-waves are the ability to analyze DICOM files directly without pre-processing or file conversion, general ease of use, ability to review, compare, quality control, and revise E-wave delineations in the program environment, semi-automatic fitting and efficient export of results in various formats. Furthermore, Echo E-waves does not require installation of other software requiring licensing costs.

## Conclusions

The software program Echo E-waves is freely available and can be used to quantify and investigate diastolic function and dysfunction using insights from classical mechanics in the PDF framework, producing results rapidly and reliably enough for application of this method for use in the cardiac imaging community.

## Appendix

Mathematical details of the PDF framework

The equation describing the balance of forces in a damped harmonic oscillator is

1 $$ m\frac{{\mathrm{d}}^2x}{\mathrm{d}{t}^2}+c\frac{\mathrm{d}x}{\mathrm{d}t}+kx=0. $$

In the PDF framework, inertia (*m*) is set to 1 g, with the other constants formulated on a per unit mass basis. The units of the other constants are g/s^2^ (N/m) for *k* and g/s (Ns/m) for *c*. In the analysis of E-waves, *x* will always be a negative number with the unit m, however as it basically describes a displacement or distance, and considering that typical values at the onset of motion (*x*_*0*_) are in the region of − 0.1 m, we have chosen to present the results of *x*_0_ as a positive number with the unit cm, for ease of reading. From the foundational equation (1), expressions describing the velocity of motion as function of time can be derived. For underdamped cases, defined by *c* ^2^ − 4 *k* < 0, the expression for velocity (*v*) as a function of time (*t*) is

2 $$ v(t)=\frac{-k{x}_0}{\omega }* \exp \left(\frac{-c}{2}t\right)* \sin \left(\omega t\right), $$

where

3 $$ \omega = \frac{\sqrt{4k-{c}^2}}{2}. $$

For overdamped cases, defined by *c* ^2^ − 4 *k* > 0, the expression is

4 $$ v(t)=\frac{-k{x}_0}{\beta }* \exp \left(\frac{-c}{2}t\right)* \sinh \left(\beta t\right), $$

where

5 $$ \beta =\frac{\sqrt{c^2-4k}}{2}. $$

For critically damped cases, defined by *c* ^2^ − 4 *k* = 0, the expression is

6 $$ v(t)=-k{x}_0t* \exp \left(\frac{-c}{2}t\right). $$

Curve fitting the appropriate function to the time-velocity data obtained from the detection of the velocity profile results in a *c*, *k*, and *x*_0_ value for each E-wave. Using these constants, the peak velocity (Vmax) can be calculated as the velocity at the time point of no acceleration or deceleration. The energy available for filling at the onset of the E-wave is ½*kx*_0_^2^ [mJ]. For underdamped cases, exact values for the deceleration time and area under the curve (velocity time integral, VTI) can be calculated, deceleration time being

7 $$ \mathrm{D}\mathrm{T}=\frac{\pi }{\omega }-\left(\frac{1}{\omega}\right)*{ \tan}^{-1}\left(\frac{2\omega }{c}\right), $$

and VTI being the integral under the curve from the starting point *t* = 0 to the time point where the velocity reaches zero.

For overdamped and critically damped cases, velocity approaches zero asymptotically and thus an exact time point where the curve returns to zero velocity does not exist. However, an approximation can be formulated resembling the manner in which the deceleration time is measured in clinical echocardiography. Shmuylovich formulated this solution for the overdamped case [13]:

8 $$ DT=\frac{1}{\sqrt{k}}*\frac{\gamma }{2{y}^2*{e}^{\gamma }-y}, $$

with

9 $$ y = \frac{c}{2\sqrt{k}}, $$

and

10 $$ \gamma = \ln {\left(\frac{1+\sqrt{1-{\mathrm{y}}^{-2}}}{1-\sqrt{1-{\mathrm{y}}^{-2}}}\right)}^{-\frac{1}{2\sqrt{1-{\mathrm{y}}^{-2}}}}. $$

The fact that each E-wave can be described in terms of three constants, with *c* corresponding to impediment to recoil in a general sense, also makes it possible to calculate an E-wave free from the energy loss which a non-zero *c* entails. Setting *c* to zero, but using the *k* and *x*_0_ values from an actual E-wave, it is possible to compare the VTI and deceleration time of an ideal E-wave free from energy loss, with those of the actual E-wave. The ratio of the actual VTI to the ideal VTI is termed the kinematic filling efficiency index (KFEI) [7]. The difference in E-wave duration between the actual and ideal E-wave is termed the relaxation component of the deceleration time (DTr), describing the prolongation of early filling due to imperfect relaxation and viscosity. DTr has been shown to have a close linear relationship with *tau* measured invasively [4].

From equation (1) the conditions at peak flow velocity (Vmax, where $\frac{{\mathrm{d}}^2x}{\mathrm{d}{t}^2}=0$ and *t* = *t*_peak_) can be stated as

11 $$ cVmax+k{x}_{t_{peak}}=0, $$

implying that these forces are equal and of opposite direction. Assuming that the relationship between the force driving recoil at the onset of flow (*kx*_0_) and at peak recoil velocity (*kx*_*t*peak_) is linear (having the form y = M’x + B’), equation (11) can be restated as

12 $$ cVmax+{M}^{\hbox{'}}k{x}_0+{B}^{\hbox{'}}=0, $$

from which follows that

13 $$ k{x}_0=M* cVmax+B. $$

Since equation (1) is applicable at all levels of load, equation (13) should be load independent as well, with *M* describing the relationship between the driving and resistive forces of filling at different levels of load; *M* can thus be termed a load independent index of filling, and its load independency has been shown in human experiments [5].

## Abbreviations

DICOM: Digital imaging and communications in medicine
KFEI: Kinematic filling efficiency index
LV: Left ventricle
PDF: Parameterized diastolic filling
PW: Pulsed Wave (Doppler)
